# Towards a Connectomic Description of the Osteocyte Lacunocanalicular Network in Bone

**DOI:** 10.1007/s11914-019-00515-z

**Published:** 2019-05-15

**Authors:** Richard Weinkamer, Philip Kollmannsberger, Peter Fratzl

**Affiliations:** 1 Department of Biomaterials, Max Planck Institute of Colloids and Interfaces, 14424 Potsdam, Germany; 2 Center for Computational and Theoretical Biology, Universität Würzburg, Campus Hubland Nord 32, 97074 Würzburg, Germany

**Keywords:** Connectome, Osteocyte network, Canaliculi, Image analysis, Image quantification, Fluid flow

## Abstract

**Purpose of Review:**

Osteocytes are the most abundant bone cells. They are completely encased in mineralized tissue, sitting inside lacunae that are connected by a multitude of canaliculi. In recent years, the osteocyte network has been shown to fulfill endocrine functions and to communicate with a number of other organs. This review addresses emerging knowledge on the connectome of the lacunocanalicular network in different types of bone tissue.

**Recent Findings:**

Recent advances in three-dimensional imaging technology started to reveal parameters that are well known from general theory to characterize the function of networks, such as network density, degree of nodes, or shortest path length through the network.

**Summary:**

The connectome of the lacunocanalicular network differs in some aspects between lamellar and woven bone and seems to change with age. More research is needed to relate network structure to function, such as intercellular transport or communication and its role in mechanosensation, as well as to understand the effect of diseases.

## Introduction

Osteocytes are the most abundant bone cells. They form a complex cell network within compact bone tissue being housed within a bone porosity consisting of canaliculi and lacunae, which form the lacunocanalicular network (LCN). While the general existence and micro-anatomy of the LCN has been known for a long time [1], it sparked renewed interest in the last years, due to the connections of the osteocyte network with other organs [2•, 3•], and its importance for phosphate metabolism. In this context, osteocytes communicate with the kidney through the factor FGF23 [4], but also with the brain through the expression of leptin [5]. Interestingly, this communication between bone and other organs has already been postulated millenaries ago in the Inner Canon of the Yellow Emperor (Huangdi Neijing), the classic text of Chinese medicine. According to this text, the human body is divided into five organ networks, one of them connecting the kidney with bone, marrow, and the brain [6]. Besides this interaction of osteocytes with other organs, the connectivity between osteocytes themselves is of crucial importance to understand their contribution to bone health. Osteocytes are regulating bone remodeling and, in particular, bone’s mechanobiological adaptation, for example, through sensing fluid flow and the expression of sclerostin [7]. This means that the osteocyte network has to function as a (mechano)sensory organ which implies a complex communication between the cells [8]. While some known characteristics of the LCN, including the average number of canaliculi emerging from lacunae, have been recently reviewed [9•, 10], the overall network characteristics and, in particular, the connectome of the network, i.e., its “wiring diagram,” received less attention. The aim of this short review is to summarize our emerging knowledge on the LCN connectome made possible by recent progress in imaging methodology. Since connectomics is already much further developed in neuroscience, the review mentions possible areas where bone researchers can learn from neuroscientists.

Conceptually, it is important to distinguish between the lacunocanalicular network (LCN) and the osteocyte network (ON) as connected cell network (see Fig. 1 in [11•]). However, most of the functions of the osteocyte network can only be understood in the interplay between the “biological” cell network and the “material” porosity in the mineralized matrix [8]. In human osteons, canaliculi that are not oriented towards the Haversian canal were found to be co-aligned with the preferred matrix orientation [12]. The pericanalicular matrix in the immediate vicinity of the cell processes was shown to be disordered [13] and also more mineralized [14•] with an increased thickness of the mineral particles [15•] incorporated in the collagen matrix. This higher mineral content around canaliculi is remarkable in the context of the osteocytes’ contribution to the calcium and phosphate metabolism. Recent evidence revived the almost forgotten idea of osteocytic osteolysis [16, 17]. Due to the high surface area of the LCN [9•] and the small distance from the LCN to almost any point in the bone matrix [18, 19•], osteocytes have easy access to the bone mineral and are able to demineralize bone [20]. The role of osteocytes as mechanosensors and orchestrators of bone remodeling depends crucially on the interplay between cell network and porous network. The fluid flow hypothesis [21, 22] assumes that mechanical loading squeezes the interstitial bone fluid through the pericellular space between the cell processes and bodies and the canaliculi and lacunae. The osteocytes then sense the shear forces caused by the fluid flow, where cell processes seem to be more mechanosensitive than the cell body [23]. The details of the fluid flow and the resulting shear forces do not only depend on the connectivity and the irregular shape of the canaliculi, but also on how the cells deform due to the flow [24] and how the cell processes are anchored on the canaliculi walls [25].

## General Remarks on Connectomics and Network Function

The highly interconnected and dense network of canaliculi has been compared to the network of neurons in the brain [26•]. For neurons, however, the function of the network (distributing signals among cells through synapses) is better known. In the field of neuroscience, describing the structure of the network in terms of neurons and their processes, as well as the synapses linking them, is seen as key for understanding higher-order functions such as sensory processing, motor output, or memory [27••]. Neural “connectomics” relates to the study of this structural and functional connectivity on multiple levels, including the connections between neurons and the interconnectedness of different brain regions. Connectomics studies in neuroscience have boosted the development of new high-end microscopy and image analysis methods in the last years [28] resulting in several recently published large-scale image datasets and cell-level connectomes of model organisms [29–31]. The obtained connectivity matrix (i.e., the description of which neurons are connected with each other, often without considering their spatial arrangement) has been successfully used to understand function in different model animals [32–35]. Additionally, a rich set of tools to quantify, visualize, and model neural networks has been developed [36]. Most of these methods are not specific to neurons and can, in principle, be adapted to other types of networks.

For the osteocyte network, the link between network structure and function is less obvious, and the spatial arrangement of the osteocyte network in the mineralized matrix cannot be neglected. Given the bulk of knowledge and methodologies available for the study of neural networks, it seems a promising research strategy to employ a “connectomics approach” to quantify differences between bones known to have a different response to loading conditions, for example, in diseased or aged organisms. Secondly, the connectomics data may be used to vet the multiple alleged functions of the osteocyte network by testing hypotheses regarding the efficiency of the network for the hypothesized functions.

## Recent Progress in Imaging the LCN Connectome

Connectomics approaches are always intimately linked with experimental techniques to image the interconnectivity. In neuroscience, depending on the level of study, a distinction is made between microscopic mapping (i.e., neuron-to-neuron mapping using, for example, tract tracing), macroscopic mapping of major fiber bundles in the brain, and functional mapping using diverse magnetic resonance imaging (MRI) or optogenetics techniques [37]. The encasement of osteocytes in the mineralized bone matrix makes it more difficult to observe cell activities. But in terms of studying the LCN connectome, the encasement is partly an advantage, since the structure is cast in solid bone. The strong contrast in electron density between the mineralized matrix and the porosity of the LCN allows employing X-ray and electron imaging techniques [38•], for example. Another possibility is to invert the electron contrast by casting the LCN (e.g., by methacrylate) and then image the casting result by scanning electron microscopy after dissolution of the bone matrix [39].

To provide a data set of the LCN which can be analyzed in terms of its connectome, one needs to overcome the typical conflict between resolution and field of view. Indeed, three requirements have to be fulfilled: (i) the LCN has to be imaged in three dimensions to map accurately connections between canaliculi. Therefore, conventional light microscopy (LM), scanning electron microscopy (SEM), and atomic force microscopy (AFM) [40] are inadequate imaging methods for the task; (ii) the resolution of the imaging method has to be high enough so that canaliculi and their connections can be reliably traced. This excludes conventional absorption-based micro-computed tomography (μCT), which remains, however, a powerful tool to study the shapes and spatial distributions of osteocyte lacunae [41]; (iii) the imaged bone volume has to be large enough to include a sufficient number of canaliculi with their interconnections. A single canaliculus with all its wall roughness has been imaged using TEM tomography [42], but this is clearly an insufficient field of view from a connectomics perspective. The resolution of X-ray tomography methods has been improved considerably [43, 44], using the intensive and partly coherent X-rays of synchrotrons. X-ray phase nano-tomography [14•, 45], and ptychographic X-ray computed tomography (PXCT) [46] were used to image the LCN with a voxel side length of 40–50 nm. An even higher resolution is possible with focused ion beam/scanning electron microscopy (FIB/SEM) [13, 47]. However, with all these high-resolution methods, the imaged bone volume typically includes a single or a few lacunae with their emerging canaliculi. Although these image data provide valuable insights in details of the LCN geometry and the surrounding matrix, it is obvious that they render a too small part of the LCN to be analyzed in terms of the connectome.

A powerful tool to image substantial parts of the canalicular network is a combination of staining and confocal laser scanning microscopy (CLSM) [15•]. The native (i.e., unembedded) bone sample is immersed in a solution containing a staining molecule like rhodamine. Confocal imaging gives image stacks with a distance of 0.3 μm, up to a depth of about 50 μm due to the opaqueness of mineralized bone. With a typical CLSM setting, an image stack corresponds to an imaged volume of approximately 7 × 10^6^ μm^3^. While the diameter of canaliculi is too small to be resolved with light microscopy, their shape can be resolved in fluorescent microscopy because the distance separating them is typically larger than the light-microscopic resolution.

Important progress was also made in the imaging of not only the lacunocanalicular network, but also simultaneously of the osteocyte network by staining their cell membrane, nucleus, and cytoskeleton. In the procedure, mice were injected intravenously with lysine-fixable dextran and sacrificed a few minutes after the injection. The stain stays in place at the canalicular walls during the decalcification of the sample. After the embedding and immunostaining of the samples, multiplexed confocal imaging can be used to image various aspects of the osteocyte structure [48•].

A label-free method whose working principle is based on a sensitivity to interfaces is third-harmonic generation (THG) microscopy [49•]. Although the image quality is currently lower compared to CLSM, an advantage of THG is that already embedded samples and archeological artifacts that do not have a LCN which is accessible to stains can still be studied in terms of the canalicular network. THG has also been used for in vivo imaging of osteocytes in a mouse calvaria model [50, 51].

## Image Analysis and Quantification of the LCN Connectome

An imaged bone volume with sufficiently many interconnected canaliculi has to run through an image analysis procedure before the LCN can be quantified in terms of its connectome. The first step in the image analysis is a binarization of the image into image voxels that belong to the LCN and the ones which do not by using either a globally or locally defined threshold value. The voxels representing the LCN are then segmented into voxels that belong to osteocyte lacunae and voxels that represent canaliculi. The segmentation criterion is naturally based on the very different “bulkiness” of lacunae and canaliculi. For the analysis of the lacunae, different frameworks have been developed which are based on a fitting of the lacuna by an ellipsoid [52, 53]. The resulting ellipsoids can then be quantified in terms of their shape (prolateness, oblateness), their orientation in relation to a pre-defined coordinate system, and their spatial relation to neighboring lacunae.

More intricate is the analysis of the canaliculi since the current imaging technology demands a choice to be made. Either the imaging resolution is high enough that the canaliculi are rendered reliably, but then the imaged volume is too small for a connectome approach. In this case, the quantification of the LCN structure can be continued in analogy to the analysis of the vascular network [54], which follows the standard analysis of the trabecular bone structure. Quantities that can be evaluated are canalicular volume fraction (Cn.V/TV) with TV the total bone volume, canalicular thickness (Cn.Th), canalicular spacing (Cn.Sp), and canalicular structure model index (Cn.SMI) [47]. Other local parameters include the average number of canaliculi emerging from a lacuna.

**Fig. 1:**
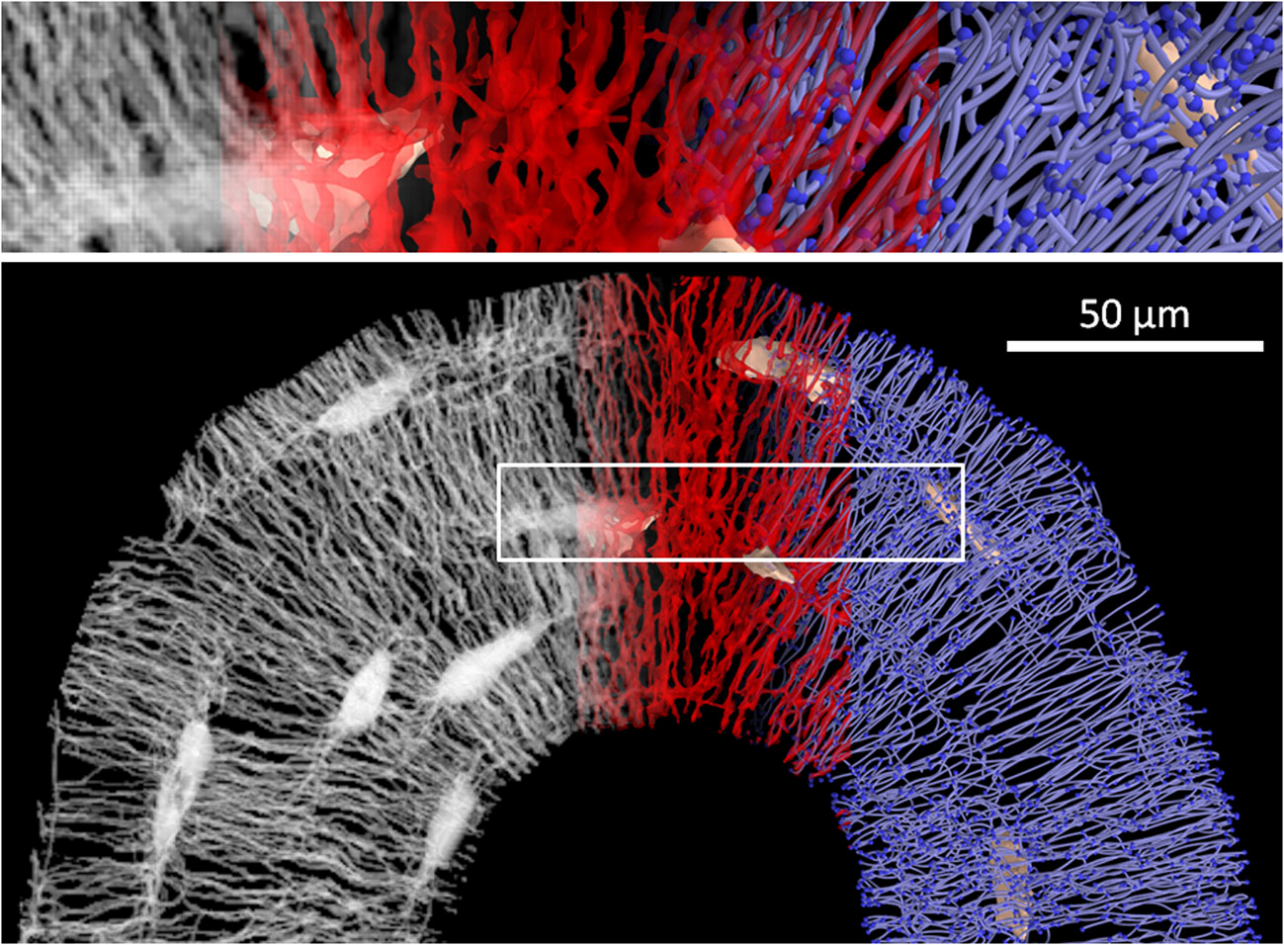
Below: work flow from an image stack obtained by confocal laser scanning microscopy (CLSM) (gray, left) to a binarized image of the LCN (red, middle) to a mathematical network consisting of edges (i.e., canaliculi) and nodes (i.e., lacunae and meeting points of canaliculi) (blue, right). The image at the top shows a magnification of the volume encircled by the white box

The alternative choice is methods which combine staining and confocal laser scanning microscopy (CLSM) (Fig. 1). In CLSM, the signal of the stain is blurred also due to penetration of the stain into the bone matrix. Attempts to quantify the canalicular volume therefore suffer from partial volume effect and easily overestimate this volume [55, 56]. However, CLSM provides reliable information on the location of the canaliculi and their interconnectivity, which can be used to infer the topological structure or “connectome” of the canalicular network (Fig. 1), although there is no information on actual cell-cell connections. To obtain the structure or “skeleton” of the LCN, the location of the canaliculi and the junctions (“nodes”) between them has to be extracted. A thinning algorithm is applied to the binarized and segmented image data to obtain the center line or medial axis of the canaliculi. In case of low signal-to-noise ratio, adaptive or topological thresholding (Kerschnitzki 2013) or machine learning with pixel classifiers or convolutional neural networks (CNN) [57] can be applied to prevent artifacts such as false negative (missing) or false positive (non-existing) links between canaliculi. Alternatively, the initial thresholding step can even be omitted by tracing canaliculi in the raw images to obtain the skeleton of the network [58]. Finally, the skeleton is converted into a mathematical network (“graph”) consisting of nodes (where at least three canaliculi meet and, therefore, including lacunae) and edges (canaliculi linking two nodes) (Fig. 1) using specialized software [18, 19•, 59].

The LCN as a network consisting of nodes and edges can be described on different levels of realistic rendering. In the most abstract form, the network is represented by a “connectivity matrix” ***A***, where each row and column corresponds to one node and the entries *a*_ij_ correspond either to the number of edges or the length of the single edge between the two nodes *i* and *j*. Since connections via canaliculi in the LCN are virtually restricted to nodes in the neighborhood of a specific node, most entries in the connectivity matrix are zero, i.e., the matrix is sparse. Alternatively, the LCN is viewed as a spatial network described by the actual position of the individual nodes and edges as curved lines in space. This representation of the LCN as a spatial network allows to tackle, for example, questions about the orientation of canaliculi towards larger structures in bones like the endosteal or periosteal surfaces or the Haversian canal in osteonal bone [12]. The close interrelation between the LCN and the surrounding bone matrix raises the question whether the network definition should be further extended for the LCN by taking parameters into account which characterize the surrounding matrix (e.g., preferred collagen matrix orientation, mineral content).

**Fig. 2:**
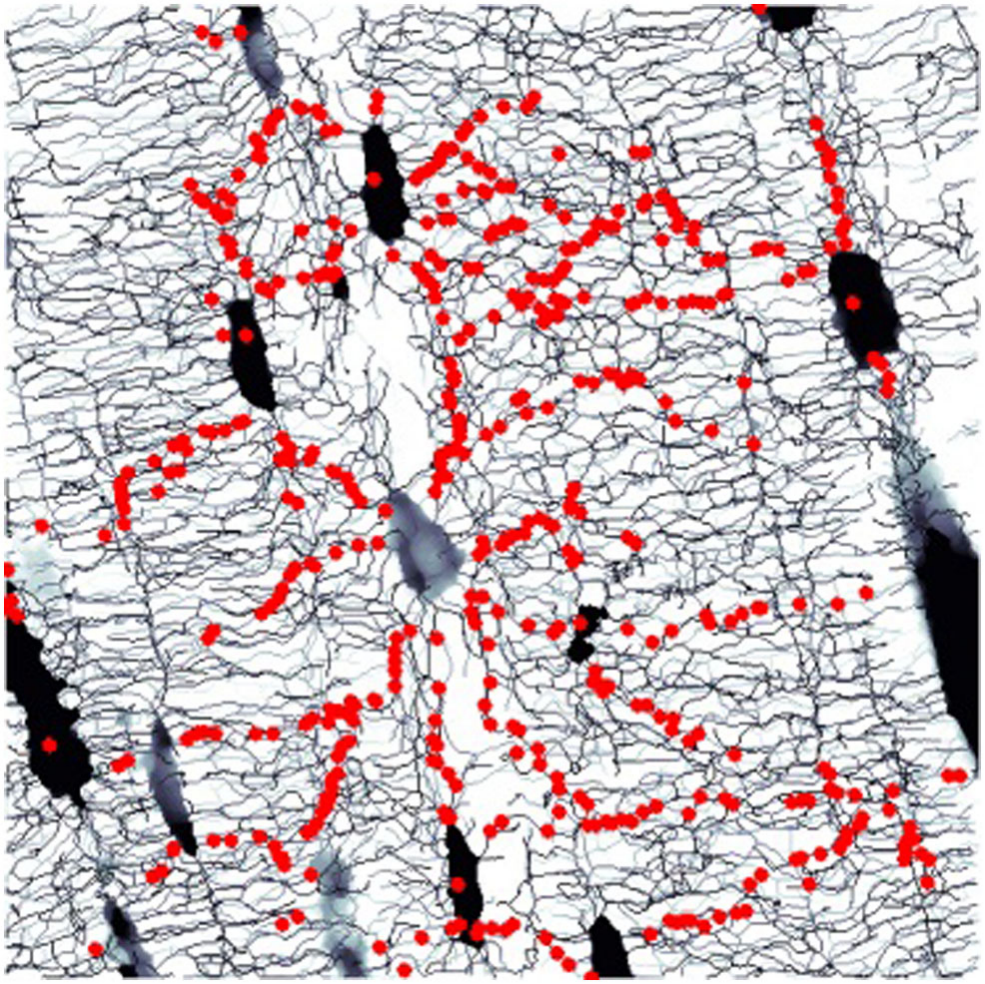
Example of a parameter of network theory applied to the lacunocanalicular network (LCN): red dots show nodes in the network with a high value of betweenness (see Table 1). These nodes line up to form “highways” through the LCN [61]

After the network structure of nodes and edges has been extracted from the images, various properties can be calculated to quantify and compare different networks (see Table 1). Since the LCN is a physical network in space, one can extract geometric measures (such as density or distances) as well as topological measures (such as node degree or clustering coefficients). The latter can be derived from the connectivity matrix [60]. The “betweenness” of a node is the number of shortest paths of the network running through that particular node. For the osteocyte LCN, nodes with high betweenness line up between cell lacunae, indicating the existence of “important” paths through the network (Fig. 2 and [61••]). The parameter of small worldness is defined as the ratio of the clustering coefficient to the average shortest path length relative to a random network and characterizes how efficient a network is in connecting distant nodes with as few edges as possible. All these parameters are well established in the field of “network science,” and a large body of existing literature allows to compare results for different types of networks, from neural networks to the internet [60, 62]. Two caveats have to be added here: firstly, generic network properties often do not account for the spatial embedding of the network [63]. For example, while in a generic network all nodes are “equivalent,” in a spatial network, long-range connections between nodes are rather unlikely. Secondly, many concepts in network research are based on simple generative models of random or regular networks. These rule-based models usually lack any biological plausibility and should be, therefore, taken with care.

**Table 1:** Selection of parameters defining the connectome of the lacunocanalicular network


Parameter		Unit
Node degree	Number of edges per node (d in the sketch)	–
Edge length	Distance between two nodes (l in the sketch) considering the tortuosity of the canaliculi	μm
Node density	Number of nodes per unit volume (cell lacunae being considered as nodes)	μm^−3^
Canalicular density	Total length of canaliculi per unit volume	μm^−2^
Lacunar density	Number of osteocyte lacunae per unit volume	μm^−3^
Distance to bone matrix	Average closest distance from any point in the bone tissue to the network	μm
Degree of edge alignment	The alignment can be defined either with respect to a fixed coordinate system (e.g., the Haversian canal) or as mutual alignment of the canaliculi	–
Clustering coefficient	= 0 if none of the neighbors of a node are linked by canaliculi, and = 1 if all possible links between neighbors of a node exist	–
Average shortest path	Global network property that denotes how many nodes have to be traversed on average to reach any node in the network from any other node	–
Betweenness of a node	Number of shortest paths of the network running through that particular node	–
Small worldness	Ratio of the clustering coefficient to the average shortest path relative to a random network	–

## LCN Connectome in Different Bone Tissues

Published results that characterize the connectome of the canalicular or the osteocyte network are rather scarce. An important reference value for the denseness of the LCN in healthy humans was established recently on a study of femoral osteonal bone. In osteons, the average canalicular density was found to be Ca.Dn = 0.074 ± 0.015 μm/μm^3^, which means that a cubic centimeter of osteonal bone contains on average canaliculi of a total length of 74 km. Assuming the canaliculi of a cylindrical shape with an average diameter of Ca.Dm = 315 nm [9, 45], this corresponds to a canalicular porosity of $\frac{\mathrm{Ca}.\mathrm{V}}{\mathrm{TV}}=\frac{D^2}{4}\bullet \pi \bullet \mathrm{Ca}.\mathrm{Dn}=0.0058$, i.e., the canalicular porosity is only slightly larger than 0.5%. For 80% of the bone, the distance to the closest canaliculus was found to be smaller than 2.8 μm. In other species, these impressive numbers were even surpassed. The highest values of the canalicular density up to now were found in the fibrolamellar bone of sheep (femur, mid-diaphysis), Ca.Dn = 0.19 ± 0.01 μm/μm^3^. In between the human and the ovine network density, the density in the particularly disordered part of murine cortical bone was determined to be Ca.Dn = 0.10 ± 0.01 μm/μm^3^. The denser network in sheep entailed that about 95% of the bone matrix was found to be within 2 μm from the network. In the same study, a network analysis showed that this dense, regularly organized network in sheep is less connected, but more efficiently organized compared to the network in irregular and fast-growing bone tissue from mice. The statistical topological properties such as edge density (fraction of possible connections between nodes that actually exist) as well as edge length and node degree distribution are identical in both network types, indicating that despite pronounced differences at the tissue level, the topological architecture of the osteocyte canalicular network at the subcellular level may be independent of species and bone type [61••].

## Influence of Disease on the LCN Connectome

Already more than a decade ago, histological observations pointed to changes in the LCN’s architecture for the most prominent bone diseases [64]. Beside the orientation and the tortuosity of the canaliculi, the connectivity of the network was altered. In particular, a reduced connectivity was observed in osteoporotic and osteoarthritic bone, while in case of osteomalacia, the LCN connectivity was not reduced [64, 65]. Since these studies were not quantitative, the statistical significance of these findings still has to be tested. Using the rat ovariectomy (OVX) model of postmenopausal osteoporosis, the rats did not display a reduced number of canaliculi emerging from the lacunae compared to the control animals, although the diameter of the canaliculi was found to be increased [55]. Using a Wistar rat model of glucocorticoid-induced osteoporosis (GIO), the difference in network architecture to control animals was rather subtle, with no statistically significant difference for the total number of canaliculi emerging from the lacuna, but with a significant difference in the number of canaliculi per surface area of the lacuna [46]. The perlecan-deficient Hypo mouse had a reduced canalicular number density, while the Akita mouse, a model for spontaneous type 1 diabetes, did not show any statistically significant changes in the network architecture [66]. Imaging the osteocyte network (ON) of osteocytes, the architecture of the network in high fat–fed diabetic mice was found to have a higher mean node degree, while the average edge length was decreased compared to lean control animals [67].

## Aging and Formation of the LCN Connectome

An important question is whether changes in the LCN architecture and connectome with age [68] contribute to age-related bone loss and to a reduction in bone’s mechanosensitivity as observed in mice [69]. A recent study on a standard C57BL/6 mouse model with mice aged between 5 and 22 months investigated the influence of age on both the osteocyte network and the lacunocanalicular network using multiplexed confocal imaging [70••]. Focusing on local connectivity of the network structures, a strong reduction in the number of cell processes, in particular in female animals, was observed with age. Also, the number of canaliculi per lacuna decreased significantly with age. While in young animals, the number of canaliculi outnumbers the number of cell processes only by a factor of 1.2–1.4, this factor increases with age to 1.5–1.7; therefore, relatively more canaliculi remain unoccupied by cell processes in older animals [70••].

Studies on human bone also indicate a reduction in the network density [56]. As human bone is an example of bone undergoing substantial remodeling during a lifetime, the distinction between the age of the individual and tissue age is crucial. The report of large volumes in human osteons without accessible LCN [19•] provides the view that an architectural degeneration of the LCN with age is a spatially heterogeneous process rather than a homogeneous thinning of the network. The observation of cell processes without cell body shows the possibility that cell remnants can still be present after cell death or apoptosis [70••]. On the long run, the absence of a living osteocyte will most likely result in micropetrosis [71] and a local clogging of the LCN.

More research is also needed to understand how the LCN is formed in the first place. In comparison between the osteocyte network of embryonic mice with mice 6 weeks of age, the embryonic mice had fewer cell processes radiating from an osteocyte than the older animals [72]. Factors that might influence network formation can be both cell-intrinsic properties as well as the tissue environment. For example, cell age and differentiation might change the dynamics of cell processes, or bone material properties could regulate their outgrowth velocity. Also, mechanical stimulation has to be considered as a potential controlling factor in the formation process of the network, where mechanical forces could not only result from muscle action, but also from changes in the osmotic pressure resulting in water-generated stresses in the collagen matrix [73]. To really understand the formation process of the LCN formation, the aim has to be to shift from the static network images available now, to dynamic movies of the formation process. Here, computer simulations can be a helpful tool [74, 75]. Based on assumptions about the local growth process, such as how often osteocyte processes branch or how they respond to tissue orientation, soluble gradients, mechanical stimulation, or physical constraints, a growing network can be simulated. The resulting network can be quantified by deriving the same properties as from images of the LCN. Subsequently, the growth rules in the model can be varied and the process iterated until the simulation results in a plausible network pattern. To confirm the findings by such computer simulations, growing networks should be imaged and quantified ideally in a time-resolved manner, for example, using live cell microscopy on short timescales, or time lapse in vivo microscopy for longer timescales.

## Conclusions

Connectomics of the osteocytic lacunocanalicular network is only starting to emerge. More research in this direction is needed to help clarifying the function of the osteocyte network. In light of the putative multifunctionality of the osteocyte network, this is a long-term perspective. As the first steps in this direction, fluid flow analyses through the LCN have been performed to understand the influence of network architecture on fluid flow–mediated mechanosensation. However, up to now, the used LCN connectomes were still highly idealized in these approaches [76–78].

A more accessible goal would be to resolve the question of whether the LCN connectome can be used as a fingerprint of different types of bone tissue. How much can we learn from a LCN connectome in terms of skeletal site, bone type, sex, and age, and does a closer look at the LCN provide new possibilities to diagnose bone diseases? If the LCN can be imaged in historical bone samples, this aspect could also be of major interest for anthropologists. On the basis of a sound characterization of the LCN connectome in different bone types, a next step could be to try to positively influence the network architecture, e.g., by applying mechanical stimulation or drug treatment.

Work on the LCN connectome can clearly benefit from other fields, such as neural or vascular networks, and the results characterizing the different connectomes could be compared to get deeper insights in how network-like structures form and are adapted for communication and transport. In particular, the comparison between the osteocyte network and the neural network seems promising, not only because of a certain similarity in the appearance of the two network structures. Neural networks possess a directionality with signals being transmitted from the presynaptic to the postsynaptic neuron and the network clearly performs a “computational” function. In bone with the presence of the two networks, the ON and LCN, with one inside the other, and the remaining space filled with interstitial fluid, the network structure seems to be even more favorable for function. In particular, osteocytes have been reported to use the LCN dynamically by expanding and contracting their cell bodies in the lacunae and moving their cell processes in and out of the canaliculi [38, 79]. With some regulation, this movement of cell processes could be used to influence the direction of the fluid flow, or even act as a system with valves, if the cell process could completely block the fluid flow within a canaliculus. If osteocytes would actively manipulate the fluid flow through the LCN in such a way, this would allow communication independent of direct cell-cell contact via gap junctions, mediated by fluid flow. This raises the interesting question whether osteocyte networks may even be solving computational tasks [22, 26], e.g., related to mechanical adaptation. Even without such speculations, the next decade of research on the connectome of the osteocyte and the lacunocanalicular network will not only help clarifying the function of the most abundant bone cell, but may likely also provide us with some surprises.
